# Environmental Determinants of Infectious Disease Transmission: A Focus on One Health Concept

**DOI:** 10.3390/ijerph15061183

**Published:** 2018-06-06

**Authors:** Hui-Yi Yeh, Kou-Huang Chen, Kow-Tong Chen

**Affiliations:** 1 Division of Cardiology, Department of Internal Medicine, Chi-Mei Medical Center, Liouying, Tainan 736, Taiwan; b891040733@yahoo.com.tw; 2 School of Mechanical & Electronic Engineering, Sanming University, Sanming City 365004, China; a0939640405@gmail.com; 3 Department of Occupational Medicine, Tainan Municipal Hospital (Managed by Show Chwan Medical Care Corporation), Tainan 701, Taiwan; 4 Department of Public Health, College of Medicine, National Cheng Kung University, Tainan 701, Taiwan

This special issue of IJERPH focuses on one health concept: we emphasize the interdependence between humans and non-human species in complex socio-ecological systems and the environmental factors affecting infectious disease transmission. As a result of improvements in sanitation and overall living conditions during the past several decades and the subsequent introduction of many vaccines and antibiotics, tremendous progress has been made in the prevention and control of infectious diseases. Globally, some infectious diseases (e.g., smallpox) have been eradicated [1]. In addition, the annual incidence of several vaccine-preventable diseases is at a low level. Despite these successes, infectious diseases remain the leading cause of death in the world [1,2]. The World Health Organization (WHO) estimates that approximately one-third (e.g., 20 million) of the annual deaths worldwide are attributed to infectious diseases [2]. The morbidity from infectious disease has increased during the past few decades and represents at least 70% of emerging infectious diseases (EID), which are a significant burden on global economic and public health [3,4,5].

Epidemics of infectious diseases threaten individuals’ lives and cause major economic losses for society, and diseases sometimes emerge and re-emerge in unpredictable regions and at unpredictable times [6,7]. The introduction of potent antibiotics and vaccines into modern medicine in recent decades inspired an overly optimistic view of our ability to eliminate or even eradicate specific infectious diseases [4,7]. This perspective has now been changed by the realization that new emerging infectious disease threats continue to occur, and old infectious diseases continue to invade different communities. Influenza, cholera, tuberculosis, malaria, dengue, and hemorrhagic fever still cause most of the illnesses and mortality worldwide, and this situation has not changed in recent years [2,4].

Emerging infectious diseases (EIDs) cause a substantial economic and public health burden in the world [8,9]. The most likely causes of the emergence of EIDs is are socioeconomic, environmental and ecological factors [7,8,9,10,11,12,13], even though there is no stricter comparative study to explicitly analyze these linkages to clarify the relationship between temporal and spatial patterns of EIDs. A previous study shows that EID events are dominated by zoonoses (60.3%), the majority of which (71.8%) originate in wildlife (e.g., severe acute respiratory virus, Ebola virus), and the morbidities are increasing significantly over time [14]. It is postulated that the origins of EIDs are significantly correlated with socioeconomic, environmental and ecological factors that provide a clue for identifying regions where new EIDs are most likely to originate [12,14]. These factors also present a basis of risk for wildlife zoonotic and vector-borne EIDs originating at lower latitudes, where reporting effort is low [14].

The inextricable interconnections among humans, domestic animals, and wildlife and their social and ecological environments are evident and require integrated approaches to human and animal health and their respective social and environment contexts [15,16]. The concept of one medicine is raised by Calvin et al. [17]. It recognizes that there is no difference in paradigm between human and veterinary medicine and that both disciplines can contribute to the development of the other. In other words, human and animal health are intimately connected. Understanding the health and disease of humans and non-humans needs a unity of approaches achievable only through a consilience of human, animal and wildlife health [15,16,17]. Considering a broad approach to the sciences related to health and well-being, the original concept of one medicine was extended through different actions and scrutinized validation in different situations [15].

‘One Health’ is an approach that considers the connections among environmental factors and the health of plants, animals, and humans [18]. Although there is much knowledge on the detection and response to infections, more public health research is needed in the field on the prediction of the emergence of infectious diseases, the range of their spread, and to improve the effectiveness and timeliness of public health responsiveness [19]. To achieve these objectives, further research is required to develop adequate laboratory skills in order to identify the pathogen genomics from more than solely one epidemiological technique that merely identifies and quantifies mechanisms for disease outbreak within a specific population. More research is also needed to develop epidemiological techniques using large databanks to identify syndromic clusters earlier. Beyond human public health, investigating the mechanisms of how animals and the environment influence the occurrence of disease is a new challenge for us. This investigation includes understanding why microorganisms cross species to cause diseases (e.g., from animals to humans), determining the factors that drive the spread of infections, and developing joint responses to outbreaks among different professional, government and international groups [19].

There are many challenges ahead. Recently, individuals worldwide have been increasingly appreciating the impact of global climate change on health [20]. The effects of climate change are complex, and it is difficult to identify the outcomes. The study outcomes include changes in the epidemiological characteristics of infectious diseases, the occurrence of zoonotic diseases, the density of vectors, the patterns of human and non-human migration, food shortages, the quality and availability of water sources, and other relevant factors [19,20]. However, we urgently need to establish epidemiological evidence on the joint effects of climatic variability and socioecological factors on the occurrence of infectious diseases in order to increase the accuracy of models for predicting the occurrence of EIDs and establishing an early warning system [20,21,22]. The development of an effective early warning system will lower the economic impact of EIDs by improving existing EID surveillance and prevention measures.

In conclusion, with complex and unpredictable patterns of emerging diseases, one of the major hurdles to designing effective control measures against infectious diseases is an insufficient understanding of the environment–agent interactions that occur with human hosts during infections. The extent to which ecological risk factors influence the risk of people contracting infectious diseases depends on the factors that increase human exposure to infected vectors (e.g., animal, mosquitos). The risk factors for humans contracting infections include the location of residence, occupation, location of leisure activity, density of transmitted vectors, and environmental factors. Future research should take into account the socioecological factors in combination with climate variables in order to gain a better understanding of the complex nature of the transmission of infectious diseases, as well as to improve the prevention and control measures for EIDs. We hope this issue will bring to light the many components surrounding EIDs as well as promote the need for future research, collaboration and innovative ideas to reduce the impact of contracting infectious diseases.
